# Association of adipocyte genes with ASP expression: a microarray analysis of subcutaneous and omental adipose tissue in morbidly obese subjects

**DOI:** 10.1186/1755-8794-3-3

**Published:** 2010-01-27

**Authors:** Robin E MacLaren, Wei Cui, HuiLing Lu, Serge Simard, Katherine Cianflone

**Affiliations:** 1 Centre de Recherche Institut Universitaire de Cardiologie et de Pneumologie de Quebec, Laval University, Quebec, G1V 4G5, Canada; 2 Experimental Medicine, McGill University Health Centre, Montreal, Quebec, H3A 1A1, Canada

## Abstract

**Background:**

Prevalence of obesity is increasing to pandemic proportions. However, obese subjects differ in insulin resistance, adipokine production and co-morbidities. Based on fasting plasma analysis, obese subjects were grouped as Low Acylation Stimulating protein (ASP) and Triglyceride (TG) (LAT) vs High ASP and TG (HAT). Subcutaneous (SC) and omental (OM) adipose tissues (n = 21) were analysed by microarray, and biologic pathways in lipid metabolism and inflammation were specifically examined.

**Methods:**

LAT and HAT groups were matched in age, obesity, insulin, and glucose, and had similar expression of insulin-related genes (InsR, IRS-1). ASP related genes tended to be increased in the HAT group and were correlated (factor B, adipsin, complement C3, p < 0.01 each). Differences between LAT and HAT group were almost exclusively in SC tissue, with little difference in OM tissue. Increased C5L2 (p < 0.01), an ASP receptor, in HAT suggests a compensatory ASP pathway, associated with increased TG storage.

**Results:**

HAT adipose tissue demonstrated increased lipid related genes for storage (CD36, DGAT1, DGAT2, SCD1, FASN, and LPL), lipolysis (HSL, CES1, perilipin), fatty acid binding proteins (FABP1, FABP3) and adipocyte differentiation markers (CEBPα, CEBPβ, PPARγ). By contrast, oxidation related genes were decreased (AMPK, UCP1, CPT1, FABP7). HAT subjects had increased anti-inflammatory genes TGFB1, TIMP1, TIMP3, and TIMP4 while proinflammatory PIG7 and MMP2 were also significantly increased; all genes, p < 0.025.

**Conclusion:**

Taken together, the profile of C5L2 receptor, ASP gene expression and metabolic factors in adipose tissue from morbidly obese HAT subjects suggests a compensatory response associated with the increased plasma ASP and TG.

## Background

Obesity is a risk factor for metabolic syndrome, cardiovascular disease, and diabetes [1,2]. The incidence of obesity has increased dramatically in the last decade throughout the world [3]. The rate of deaths from obesity related diseases is also on the rise. Understanding the factors that contribute to obesity related diseases is crucial to help obese patients achieve better health status.

Notwithstanding the strong associations of obesity with dyslipidemia and insulin resistance, it has been recognized in recent years that not all forms of obesity are the same. Visceral obesity presents a greater risk for obesity related disease than subcutaneous obesity [4]. Classification of obese populations based on their level of insulin sensitivity identifies distinct subsets variously referred to as insulin-sensitive obese [5-7], metabolically-healthy but obese [8], and metabolically-normal obese [9]. In each of these cases, specific parameters, such as glucose or insulin, are within the normal healthy range found in non-obese subjects. Most studies classify "healthier obese subjects" according to insulin sensitivity, but also according to other parameters such as HDL cholesterol, plasma triglycerides [9,10], C-reactive protein, interleukin-6, LDL cholesterol, and visceral fat [11]. We have recently shown that intracellular insulin signalling pathways in adipose tissue are different between insulin sensitive versus insulin resistant obese subjects [12].

However, in addition to altered glucose metabolism, dyslipidemias, such as elevated plasma triglyceride (TG), are common in obesity and are an independent risk factor for many diseases including diabetes, metabolic syndrome, and cardiovascular disease [1]. When coupled with other risk factors such as high LDL cholesterol, low HDL cholesterol, or insulin resistance, the risk for these diseases increases [13,14]. Accordingly, in the present study, obese subjects were evaluated based on characterization according to fasting triglyceridemia and acylation stimulating protein (ASP) to examine potential differences in adipose tissue gene expression.

Cellular studies have demonstrated that acylation stimulating protein (ASP) is a main anabolic stimulator of TG storage in adipose tissue and is produced by adipocytes [15]. ASP stimulates TG synthesis via the ASP receptor, C5L2, a seven transmembrane G protein coupled receptor [16,17]. Downstream several key signalling proteins have been identified, including phospholipase C, phosphatidylinositol-3 kinase, Akt and protein kinase C [18] culminating in increased glucose transport and diacylglycerol acyltransferase activity [18]. ASP is identical to C3adesArg and is produced through the interaction of the precursor protein C3, Factor B, and adipsin (a.k.a. Factor D), components of the alternative complement immune pathway which are secreted by adipose tissue.

In the adipose tissue milieu ASP levels increase postprandially, although general circulating levels change little [19-21]. Overall, fasting ASP is strongly predictive of postprandial TG (evaluated as area-under-the-curve: AUC) in both men and women [22]. ASP is increased in obesity, as well as insulin resistance, diabetes, cardiovascular disease, hyperthyroidism and polycystic ovary syndrome [15,23-25], all disorders associated with obesity [26,27]. However obesity is not an essential feature of elevated ASP levels, as ASP is increased in subjects with diabetes or polycystic ovary syndrome, even in the absence of obesity [25].

A direct association between plasma ASP on one hand and the metabolic function of the subcutaneous and omental adipose tissue on the other hand are unknown, and this is the first study to examine this. Further there are no studies on C5L2 adipose expression in humans. Our hypothesis was that morbidly obese subjects separated based on the fasting plasma levels of ASP and TG into HAT (High ASP and Triglyceride) and LAT (Low ASP and Triglyceride) groups would demonstrate differential expression of genes in lipid metabolic and inflammatory biologic pathways. Characterization of subcutaneous and omental adipose tissue using a microarray approach was used. To target lipid metabolic and inflammatory biologic pathways, all known genes in these pathways were identified and all those available in the microarray were pooled a priori, and then expression in LAT vs HAT for both subcutaneous and omental adipose tissue was evaluated.

## Results

### Subject Characteristics

A total of 11 subjects participated in the microarray study. A fasting blood sample and subcutaneous and omental adipose tissue samples were taken from each subject. Of the 21 adipose samples, 20 were paired omental (OM) and subcutaneous (SC) samples from 10 subjects, and the remaining SC sample was unpaired. As shown in Table 1, the subjects were divided into 2 groups based on an ASP-TG lipemic index (as described in methods). Using this functional ASP calculation two groups were defined, one with low ASP, low TG, and thus a lower lipemic index which we have termed the Low ASP and TG group (LAT, n = 4) and a second group with high ASP (60.5 ± 11.7 vs. 36.1 ± 7.4 nmol/L), TG (2.01 ± 0.2 vs. 1.3 ± 0.1 mmol/L), and lipemic index (119.5 ± 22.2 vs. 44.8 ± 5.7, p < 0.05), which we have termed the High ASP and TG group (HAT, n = 7). Both groups were morbidly obese based on body mass index (BMI) where a BMI > 30 kg/m^2 ^is considered obese and BMI > 40.0 kg/m^2 ^is morbidly obese [28]. The two groups did not differ significantly in average BMI (52.0 ± 5.8 vs. 56.4 ± 4.7, p=ns), nor in average age. Average insulin and glucose values were the same between the two groups (Table 1, p ns for all). While none of the patients had been previously diagnosed as diabetic, at the time of pre-operative sampling one subject in the LAT group and two subjects in the HAT group had glucose values > 7.0. For all tables and figures, the data are presented as averages ± SEM for the groups as indicated.

**Table 1:** Subject Characteristics.

Group	LAT	HAT	P
Age	42.2 ± 5.1	42.6 ± 3.6	ns

BMI	52.0 ± 5.8	56.4 ± 4.7	ns

ASP	36.1 ± 7.4	60.5 ± 11.7	0.05

TG	1.29 ± 0.1	2.01 ± 0.2	0.05

Lipemic index	44.8 ± 5.7	119.5 ± 22.2	0.04

Glucose	6.24 ± 1.97	6.22 ± 0.79	ns

Insulin	414 ± 67	384 ± 64	ns

Age (years), body mass index (BMI, kg/m^2^), fasting plasma ASP levels (nmol/L), fasting plasma triglyceride (TG, mmol/L), lipemic index (TG * ASP), fasting plasma insulin levels (pmol/L) and fasting plasma glucose levels (mmol/L) for Lower ASP and TG (LAT, n = 4) and Higher ASP and TG (HAT n = 7) subjects. Data (means ± SEM) are analysed by t test, where p ns indicates not significant.

### Microarray Analysis

In order to avoid sample bias, no samples were pooled prior to microarray analysis, and all 21 tissue samples were analysed individually. Two different analysis paradigms were used for the evaluation of the microarray results: (i) overall evaluation using SAM analysis and (ii) evaluation targeting the specific biological pathways of interest, lipid metabolism and inflammation. As the primary hypothesis was to evaluate LAT (Low ASP-TG index) vs HAT (High ASP-TG index) we first compared these two groups using overall analysis. The cDNA microarrays contained a total of ~20,000 probes (~10,000 human genes, plus ESTs, housekeeping genes, and positive and negative controls) which were analysed for all 21 samples. Information on Codelink Uniset 20K I Gene List #30019 can be obtained directly from http://www.gehealthcare.com/usen/microarrays/codelink_genelists.html. The complete data set is reported in GEO (Gene Expression Omnibus) available at: http://www.ncbi.nlm.nih.gov/geo/ as Accession # GSE 15524.

SAM (Significant Analysis of Microarrays) analysis was performed on SC and OM samples separately, but using the same analysis parameters. The SAM procedure [29] using version 3.09 is available at http://www-stat.stanford.edu/~tibs/SAM using suggested guidelines. This program is a validated statistical technique for identifying differentially expressed genes across high density microarrays. With an estimated false discovery rate (FDR) of 2.0%, and a minimum of a 2-fold change considered significant, 464 genes were identified as significantly different between HAT and LAT in SC tissue (which represents 2.3% of all genes on the microarray). Using the same analysis parameters, no genes were identified as significantly different between HAT and LAT in OM tissue. Using less stringent criteria (FDR = 5% and minimum 2-fold change), 1236 genes were identified in SC, but again, none in OM tissue. Even when criteria were relaxed to an FDR of 15%, only 6 genes were identified in OM. Thus, the major changes between HAT and LAT were restricted to SC adipose tissue. When gene expression in OM tissue was compared to SC tissue using paired analysis and stringent parameters (FDR = 2.0% and minimum 2 fold difference), only 108 genes were identified. Using less stringent criteria (FDR = 5%, minimum 2 fold change), only 178 significant genes were identified. By far, the major differences in gene expression in relation to the classification of subjects as HAT or LAT using the lipemic index (plasma ASP and TG) in this sample set are in SC adipose tissue. Therefore further analysis was focused on SC tissue, although supplementary information is provided for omental tissue as well.

The genes identified in SC adipose tissue were further examined. Of those genes identified as different using FDR = 2.0% and a minimum twofold change, 453 were up-regulated (positive genes) and 11 were down-regulated (negative genes) in HAT subjects as compared to LAT with q values for the genes identified ranging from 0.00000 to 0.02103. Gene description and access to Codelink Uniset 20K I Gene List #30019 http://www.gehealthcare.com/usen/microarrays/codelink_genelists.html were used to assign the genes manually into functional categories. Many of the genes identified (~50%) were tagged as EST, "hypothetical proteins" or clones labelled based on putative protein sequence as "similar to" or "hypothetical" with no known function. Of the genes with assigned function, the positively regulated genes coded for proteins involved in extracellular signalling (including cell-cell signalling and endocrine functions), intracellular signalling, intermediary metabolism and energy metabolism, structural proteins, transcription and translation (including cell growth, differentiation, apoptosis and proteolysis) based on manual group assignment. A supplementary table listing the positive and negative genes is available upon request.

As the specific aim of the study was to evaluate the biological pathways of storage lipid metabolism and inflammation, areas of current interest in adipose tissue biology, a specific analysis was then undertaken of pathways of interest in SC adipose tissue. First, housekeeping genes (as controls) were identified, then genes potentially involved in ASP and C5L2 function were identified. As ASP represents a crossroad of immune and lipid metabolism, targeted pathways included all genes for proteins that could be identified in ASP generation, complement related factors, storage lipid metabolism including TG synthesis, lipolysis and oxidation, adipocyte differentiation, and inflammatory genes. The method of analysis was the following: (i) all functional genes within the identified biologic pathways were listed; (ii) accession numbers were identified using Entrez Gene http://www.ncbi.nlm.nih.gov/; (iii) the Codelink gene list was screened to tag all of the accession numbers present in the array; (iv) all genes identified, and the associated SC and OM datasets were then pooled before initiating statistical analysis and (v) the data was then analyzed for HAT vs LAT. Using this approach where biologic pathways were identified a priori followed by subsequent statistical analysis, of the 88 functional genes identified (excluding housekeeping genes), 51 were differentially expressed in relation to HAT vs LAT (Additional File 1, Table S1), either increased or decreased, which represents 58%. A number of these genes had also been identified using the SAM analysis (such as genes involved in lipid synthesis); this is a substantial pathway enrichment.

### Housekeeping Genes & Insulin Related Genes

The expression of 4 housekeeping genes suggested for studies in adipose [30] was evaluated across all 11 SC samples was evaluated (Additional File 2, Table S2). The expression of B2M, GUSB, PPIA, and TFRC were constant across all 11 samples with no significant differences when LAT and HAT were compared. There were no significant correlations between any of the housekeeping genes and age, BMI, or fasting plasma levels of ASP, TG, insulin, or glucose.

The expression of insulin receptor (InsR), insulin receptor substrates 1 and 4 (IRS1 and IRS4), insulin like growth factors 1 and 2 (IGF1 and IGF2) were also evaluated (Additional File 2, Table). The expression of these genes was also constant across all 11 samples and not significantly different between the two groups, which is consistent with the plasma data for insulin and glucose. There were no significant correlations between these genes and age or BMI. As the two groups have similar insulin related gene profiles, differences between the 2 groups are not likely explained by differences in insulin signalling or sensitivity.

### ASP Generation Genes

**Figure 1:**
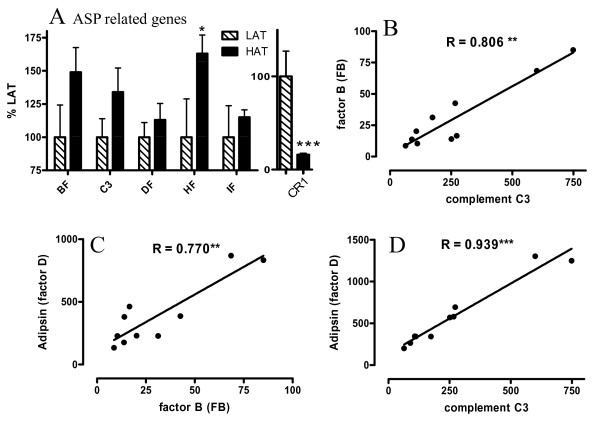
**ASP generation related genes tend to be increased in HAT group**. (A) Expression of genes related to ASP generation in SC adipose tissue from LAT (hatched bars) and HAT (solid bars) as assessed by microarray where LAT expression is set at 100%. Correlations are shown between (B) C3 and factor B (FB), (C) FB and adipsin (Factor D), and (D) C3 and adipsin in SC adipose tissue from both LAT and HAT subjects. Data is expressed as means ± SEM where * p < 0.025, ** p < 0.01, *** p < 0.001, where R values represent Spearman coefficients, and lines are based on linear regression.

We next looked at genes related to plasma ASP generation by adipose tissue. As previously published [31,32], the key genes in the conversion of complement C3 to its ASP form (aka C3adesArg) are all produced by adipocytes: C3, factor B (FB), adipsin (or factor D), and carboxypeptidase N (CPN1). Additionally, factors that enhance or inhibit the process are also produced by adipocytes: factors H, I, and properdin [33]. As shown in Figure 1A, C3, factor B and factor H are all moderately increased in HAT vs LAT (where LAT is set as 100%). We also analyzed the expression pattern of CR1, aka CD35, an inhibitor to both the classical and alternative complement pathways. CR1 inhibits both C3 and C5 convertases, thus decreasing production of C5a, C3a and desArg forms including ASP, and has a high affinity for C3b [34]. As CR1 was decreased in HAT SC tissue (Figure 1A) this reduction may be permissive for increased ASP production via C3 convertase. Properdin, which stabilizes C3 convertase, also tended to be increased (Additional File 1, Table S1). Additionally, C3, adipsin and factor B were all closely correlated to one another in SC tissue (Figure 1B to 1D). These 3 genes also correlated closely with other genes and have been termed the "ASP triad" in the present study. In omental (OM) tissue, although expression of C3 and factor B were higher than in SC, overall there was no difference between LAT and HAT for any of the factors other than a decrease in factor B in HAT subjects (Additional File 1, Table S1).

### Real time RT-PCR Analysis of ASP Receptor C5L2

**Figure 2:**
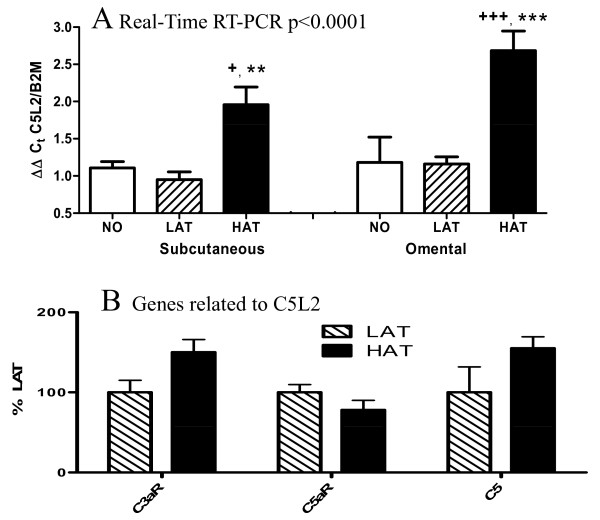
**C5L2 expression is increased in HAT group while similar immune receptors are unaltered**. (A) C5L2 gene expression in SC and OM adipose tissue from control non-obese (NO), LAT, and HAT subjects as assessed by ΔΔ real time RT-PCR for C5L2 relative to the housekeeping gene B2M. 2-way ANOVA indicates significant differences (p < 0.0001) with a difference between groups (p < 0.0001) but no significant difference between tissues (pNS). Post-hoc test indicates that HAT was significantly increased vs LAT in SC (p < 0.0.01) and OM (p < 0.001), and HAT was significantly increased vs NO (non-obese) in both SC (p < 0.05) and OM ((p < 0.001) where * p < 0.05, ** p < 0.01 and *** p < 0.001 for HAT vs LAT and + p < 0.05, ++ p < 0.01 and +++ p < 0.001 for HAT vs NO. There were no significant differences between LAT and NO. (B) Gene expression of immune receptor genes related to C5L2 in SC adipose tissue from LAT (hatched bars) and HAT (solid bars) as assessed by microarray where LAT expression is set at 100%. No significant differences between the groups were found for any of the related receptors. Data is expressed as means ± SEM.

As C5L2, a newly identified receptor, was not present on the microarray used for analysis, quantitative expression of C5L2 in the LAT and HAT groups was measured using TAQMAN real time RT-PCR via the delta-delta Ct method relative to expression of B2M, a housekeeping gene. For the real time RT-PCR, a non-obese control group was added and results are provided for both SC and OM tissue, as this is the first quantitative measurement of C5L2 in human adipose tissue. This additional non-obese group had the following characteristics: BMI 25.1 ± 1.4 kg/m^2^, age 54.8 ± 7.6 years, plasma ASP 30.4 ± 1.5 nmol/L, plasma TG 1.12 ± 0.18 mmol/L, glucose 3.75 ± 0.21 mmol/L, insulin 137.2 ± 16.1 pmol/L and lipemic index 34.8 ± 6.7. As shown in Figure 2A, using 2-way ANOVA, there is a significant difference in groups (p < 0.0001), but no significant difference between tissues (SC vs OM). With post-hoc analysis, HAT is significantly increased vs LAT in SC and OM (p < 0.01 and p < 0.001, respectively), and HAT is also significantly different from NO in SC and OM (p < 0.05 and p < 0.001, respectively), while there was no significant difference between LAT and NO. There was also a positive correlation between expression in SC adipose tissue and OM adipose tissue, such that subjects with high expression in SC tissue also had high expression in OM (R = 0.676, p = 0.023).

### Other complement factors and receptors

The majority of the genes involved in ASP production were initially identified as being alternative complement factors and thus part of the immune system [35]. Therefore we looked at the regulation of other complement factors, including C5 and two receptors of complement activation, C3aR and C5aR. The sequence of C5L2 closely resembles these two receptors. It has been demonstrated [16,17,36] that C3a, C5a, and ASP all bind to C5L2, only C3a binds to C3aR, and only C5a binds to C5aR. Both C3aR and C5aR are expressed at similar levels in the LAT and HAT groups for both SC and OM tissues (Figure 2B and Additional File 1, Table S1) and there is no significant correlation with C3, factor B or adipsin, although C5 had a negative correlation.

### Lipid Synthesis Genes

**Figure 3:**
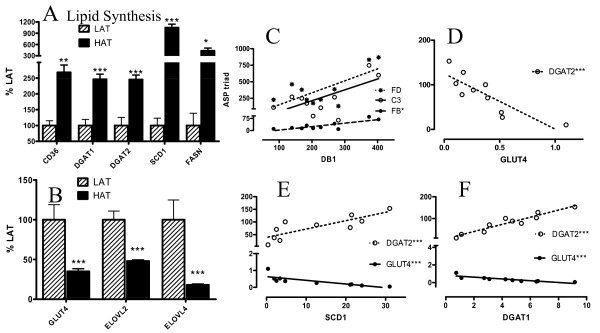
**Intracellular lipid synthesis genes in HAT vs LAT in SC adipose tissue**. Gene expression of intracellular lipid synthesis genes (A and B) in SC adipose tissue from LAT (hatched bars) and HAT (solid bars) as assessed by microarray where LAT expression is set at 100%. Correlation in SC adipose tissue from both LAT and HAT subjects between the ASP triad of genes (C3, factor B and adipsin) and (C) DB1 presented for C3 (open circles), FB (solid circles) and adipsin (stars). R values for C3, FB, and FD respectively are (C) 0.548, 0.639, 0.509. (D) Correlations between DGAT2 and GLUT4, R = -0.891. Correlations between GLUT4 and DGAT2 and (E) SCD1 and (F) DGAT1 shown as GLUT4 (solid circles) and DGAT2 (stars). R values (Spearman coefficients) for GLUT4 and DGAT2 respectively are (E) -0.952, 0.903 and (F) -0.964, 0.952. Lines are based on linear regression. Data is expressed as means ± SEM where * p < 0.025, ** p < 0.01 and *** p < 0.001.

A wide variety of genes are involved in dietary lipid storage and de novo lipid synthesis. We have focused here on all of the key genes that could be identified from each pathway including fatty acid and glucose transporters (CD36, GLUT4), and TG synthesis enzymes such as diacylglycerol acyltransferases (DGAT1 and DGAT2), and fatty acid elongating enzymes (ELOV) potentially related to ASP function. Microarray analysis revealed increased expression of fatty acid transporter (CD36) but decreased glucose transporter (GLUT4, Figure 3). Genes involved in TG synthesis were up-regulated in the HAT group, including stearoyl CoA desaturase (SCD1), DGAT1 and DGAT 2, fatty acid synthase (FASN), phosphatidic acid phosphatase (PAP2A2) and malic enzyme (ME1) (Figure 3, Additional File 1, Table S1). On the other hand, other genes related to de novo fatty acid synthesis were down-regulated (Figure 3B). The fatty acid elongation enzyme genes (ELOVL2 and ELOVL4) were both down-regulated (Figure 3B). By contrast there were no significant differences in OM tissue, other than an increase in ME1 in HAT subjects (Additional File 1, Table S1). It is interesting to note that there is a significant correlation with the ASP triad (C3/B/adipsin) and specific lipid metabolic genes, where DB1, DGAT1, ME1 and Glut4 are examples (Figure 3 and Additional File 1, Table S1) Several other genes correlated well within the group of metabolic genes such as DGAT1, DGAT2, SCD1, FASN, GLUT4 and ELOV2 (Figure 3 and Additional File 1, Table S1).

### Lipolysis Related Genes

**Figure 4:**
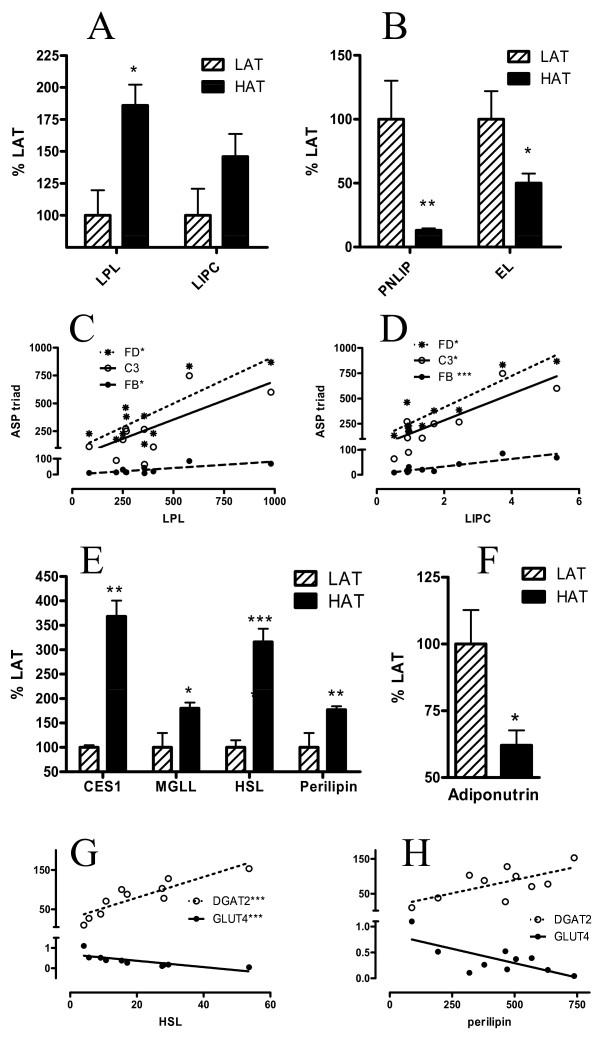
**Expression of Lipolytic genes in LAT vs HAT**. Gene expression of lipolytic genes (A) LPL and LIPC, (B) PNLIP and EL and (E) CES1, MGLL, HSL, perilipin and (F) adiponutrin in SC adipose tissue from LAT (hatched bars) and HAT (solid bars) as assessed by microarray where LAT expression is set at 100%. Correlation between the ASP triad of genes and (C) LPL and (D) LIPC are shown for C3 (open circles), FB (solid circles), and adipsin (stars). R values (Spearman coefficients) for C3, FB, and adipsin respectively are (C) 0.430, 0.600, 0.564 and (D) 0.673, 0.842, 0.709. Correlations between lipid metabolism genes GLUT4 and DGAT2 with (G) HSL and (H) perilipin are shown for GLUT4 (solid circles) and DGAT2 (stars). R values (Spearman coefficients) for GLUT4 and DGAT2 respectively are (G) -0.952, 0.915 and (H) -0.564, 0.515. Lines are based on linear regression. Data is expressed as means ± SEM where * p < 0.025, ** p < 0.01 and *** p < 0.001.

A number of intracellular and extracellular adipocyte lipases hydrolyze TG. The main extracellular lipase, lipoprotein lipase (LPL), hydrolyzes dietary fats, releasing fatty acids for cellular transport and intracellular esterification to TG. LPL and hepatic lipase (LIPC), both of which localize to the cell surface membrane, tended to be up-regulated in the HAT group vs LAT and correlated significantly with the ASP triad in SC tissue (Figure 4A, 4C, 4D). On the other hand, two additional extracellular lipases, pancreatic lipase (PNLIP) and endothelial lipase (EL), were down-regulated (Figure 4B and Additional File 1, Table S1).

Hormone sensitive lipase (HSL) was considered the primary intracellular adipocyte lipase responsible for releasing fatty acids from the cell; however, additional lipases have subsequently been identified. Both HSL, and perilipin, which interacts directly with HSL to regulate lipolysis, were up-regulated in the HAT group (Figure 4E) as were carboxylesterase 1 (CES1, aka triacylglycerol lipase) and monoglyceride lipase (MGLL) (Figure 4E). These lipases correlated closely with the metabolic genes GLUT4, DGAT2 and HSL (Figure 4G, 4H, Additional File 1, Table S1). Adiponutrin, on the other hand, was down-regulated in the HAT group (Figure 4F). Again, there were no differences for any of the genes in OM tissue, other than a decrease in LIPC in HAT subjects (Additional File 1, Table S1).

### Oxidation Genes

A number of genes related to oxidation in adipose tissue were down-regulated in HAT vs LAT (Additional File 1, Table S1), including carnitine palmitoyltransferase (CPT-1C), carnitine acetyltransferase (CRAT), uncoupling protein (UCP1), AMP-activated kinase (AMPK) as well as some AMPK regulatory genes (PRKAB2, PRKAG3, Additional File 1, Table S1). Other AMPK regulatory genes (PRKAA1, PRKAG1, and PRKAG2) were up-regulated in the HAT group (Additional File 1, Table S1). Acetyl CoA carboxylase (ACC) was increased in SC HAT tissue, and had some weak correlations with the ASP triad (Additional File 1, Table S1). The contrasting regulation of ACC and AMPK is reflected in their opposing physiological roles: ACC catalyzes the rate limiting step in fatty acid synthesis and AMPK senses energy availability and inactivates ACC. CPT-1C and UCP1 (Additional File 3, Figure S1) were both correlated with GLUT4 and DGAT2 but, consistent with the trends described above, only partially with any of the genes from the ASP triad (Additional File 1, Table S1). There were no differences in OM tissue between LAT and HAT for any genes except for PRKAG1 which increased in HAT subjects (Additional File 1, Table S1).

### Fatty Acid Binding Protein (FABP) Genes

As shown in Additional File 4, Figure S2, some of the FABP group of genes were up-regulated in the HAT SC tissue, but not all (Additional File 4, Figure S2). Several correlated strongly with the ASP triad (Additional File 4, Figures S2C to S2E). Although FABP4 was first identified in adipose tissue [37], all of these binding proteins are ubiquitously expressed. Further, FABP2 and FABP7 are decreased (Additional File 4, Figure S2B). The roles of FABP are varied, including differential binding of various fatty acids, with functional links that include signalling and association with transcription factors, channelling to lipid synthesis vs oxidation, cell growth, proliferation and differentiation [38]. These differential roles of FABP may explain the opposite changes. However no differences were detected in OM tissue (Additional File 1, Table S1).

### Differentiation Genes: the C/EBP and PPAR families

**Figure 5:**
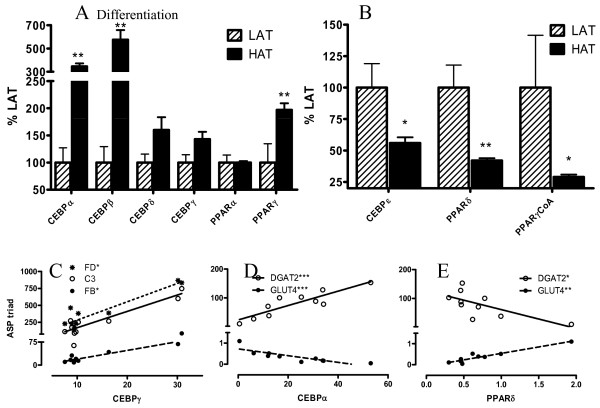
**Expression of CEBPs and PPARs in LAT vs HAT**. Gene expression of differentiation related gene families, CEBPs and PPARs (A and B) in SC adipose tissue from LAT (hatched bars) and HAT (solid bars) as assessed by microarray where LAT expression is set at 100%. Correlations between the ASP triad and CEBPγ are shown in (C) with R values (Spearman coefficients) for C3, FB, and adipsin are 0.527, 0.709, and 0.698 respectively. Correlations for CEBPα (D) and PPARδ (E) with GLUT4 and DGAT2 respectively are (D) -0.915, 0.830 and (E) 0.758, -0.673. Lines are based on linear regression. Data is expressed as means ± SEM where * p < 0.025, ** p < 0.01 and *** p < 0.001.

Both the C/EBP and the PPAR nuclear receptor families are important factors in adipocyte differentiation. The C/EBP genes α and β were increased (Figure 5), while C/EBPε was decreased (Figure 5B). C/EBPβ is a checkpoint for differentiation and is induced by C/EBPα, which is increased early in the differentiation process. Interestingly, C/EBPγ correlates with the ASP triad (Figure 5C).

PPARγ is best recognized as a late marker of differentiation. PPARγ was increased in the HAT group, along with many of the other differentiation genes (Figure 5A). However, PPARδ was an exception, being significantly down-regulated (Figure 5B). While the role of PPARδ is not fully understood, it is believed that activation of PPARδ leads to increased fatty acid metabolism [39]. In accordance with decreased expression of genes in fatty acid oxidation, and increased expression of fatty acid storage genes, the decrease in PPARδ is consistent with that interpretation. The majority of differentiation genes correlate with the metabolic genes (Figure 5). There was no change in OM tissue for any of the genes examined (Additional File 1, Table S1).

### Inflammatory Genes

Recently, there has been intense interest in the potential interactions between adipocytes and macrophages within the adipose tissue milieu. The potential role of ASP in both the immune system and storage lipid metabolism makes it all the more pertinent to examine pro-and anti-inflammatory genes in association with ASP and the lipemic index. The anti-inflammatory genes transforming growth factor beta 1 (TGFB1) and tissue inhibitor of metalloproteinase (TIMP1, 3 and 4) were increased in the HAT group (Additional File 5, Figure S3A), with decreases in some interleukin genes such as IL4. Of the pro-inflammatory interleukin and receptor genes, there was little difference between the LAT and HAT groups other than for PIG7 (Additional File 5, Figure S3B) with no difference in OM tissue (Additional File 1, Table S1).

Hand in hand with inflammation of adipose tissue, is remodelling of adipose tissue. Key genes in this process are macrophage recruitment genes and matrix metalloproteinases [40,41]. A number of genes for macrophage recruitment tended to be increased in the HAT group in subcutaneous tissue, especially macrophage migration inhibitory factor (MIF) and chemokine (C-C motif) receptor 2 (CCR2) genes which were also closely correlated with the ASP triad of genes (Additional File 6, Figures S4A-S4C). Of the matrix metalloproteinases, MMP20 and MMP10 were significantly down-regulated in the HAT group (Additional File 6, Figures S4D) while MMP 2 was increased. OM tissue had no significant changes between HAT and LAT (Additional File 1, Table S1).

### Confirmatory Analysis by Real Time PCR

Confirmatory analysis by real time PCR using the delta-delta Ct method relative to expression of B2M, a housekeeping gene, was also conducted for 7 additional genes. The genes chosen included two genes with markedly increased expression in HAT vs LAT (CD36 and DGAT2), two genes with moderately increased expression in HAT vs LAT (C3 and CEBPγ) and two genes with little change in expression in HAT vs LAT (C5aR and FABP4). In addition, results for insulin receptor (INSR) were also presented. Further, all genes were analyzed in both SC and OM tissue, for all samples available, in duplicate or triplicate. As can be seen in Table 2, overall results from the real-time PCR correlated well with the results from the microarray analysis in both SC and OM tissues from LAT and HAT subjects.

**Table 2:** Real Time PCR Analysis of Selected Genes in Subcutaneous and Omental LAT and HAT Groups

Gene	Accession #	Subcutaneous			Omental		
		**LAT**	**HAT**	**%**	**LAT**	**HAT**	**%**

C5L2	NM_018485	0.950 ± 0.105	1.958 ± 0.237**	206% (NA)	1.161 ± 0.096	2.684 ± 0.263**	231% (NA)

C3	NM_000064	0.588 ± 0.086	0.928 ± 0.117*	158% (134%)	2.655 ± 0.829	2.344 ± 0.026	88% (45%)

C5aR	NM_00173	1.412 ± 0.189	1.588 ± 0.120	112% (78%)	1.738 ± 0.240	1.926 ± 0.229	111% (122%)

CD36	NM_000072	0.877 ± 0.134	1.717 ± 0.212**	196% (268%)	0.411 ± 0.072	0.874 ± 0.103**	213% (96%)

DGAT2	NM_032564	1.671 ± 0.358	3.383 ± 0.513*	202% (245%)	0.606 ± 0.207	1.110 ± 0.151*	183% (144%)

FABP4	NM_001442	0.732 ± 0.093	0.776 ± 0.075	106% (128%)	0.638 ± 0.064	0.739 ± 0.083	116% (92%)

CEBPγ	NM_001806	1.006 ± 0.226	1.497 ± 0.126*	160% (143%)	3.371 ± 1.694	3.607 ± 1.318	97% (75%)

INSR	X02160	1.553 ± 0.278	1.580 ± 0.252	102% (112%)	1.407 ± 0.617	1.630 ± 0.371	116% (62%)

Gene expression in SC and OM adipose tissue from Lower ASP and TG (LAT) and Higher ASP and TG (HAT) subjects as assessed by ΔΔ real time RT-PCR relative to the housekeeping gene B2M. Data are means ± SEM (n = 8 to 16 total per group) and are analysed by t test, where * p < 0.05, and ** p < 0.01. Percentage change of HAT vs LAT is provided, with the comparative value from microarray analysis (see Figures and Additional File 1, Table S1 for data) given in parentheses, where NA = not available.

## Discussion

Adipose tissue has been well documented as an endocrine organ in the last two decades [42]. The recognition that ASP (aka C3adesArg) is produced by adipose tissue, and that the three precursor proteins C3, factor B and adipsin, all increase with differentiation of adipocytes [43] was one of the first demonstrations that hormones with both paracrine and endocrine functions were produced within the adipose milieu. The primary function of ASP in adipocytes appears to be promotion of lipid storage, and this is achieved through increases in fatty acid esterification and glucose uptake [44].

A number of human studies have demonstrated positive associations between ASP and various metabolic disturbances (diabetes, metabolic syndrome, cardiovascular disease, nephrotic syndrome, polycystic ovary syndrome) with or without the presence of obesity. Previous studies have demonstrated that circulating plasma ASP is associated with TG and fatty acid levels [15], with a strong correlation between fasting ASP and postprandial TG clearance even in non-obese individuals [22]. The demonstration of direct adipose tissue production of ASP postprandially in human studies [19-21], coupled to evidence that dietary TG in the form of chylomicrons directly stimulate C3 and ASP production in cultured human adipocytes [45,46] provides a strong link between plasma ASP and TG. Consequently, the aim of the present study was to evaluate microarray expression in subcutaneous vs. omental adipose tissue in relation to plasma ASP and TG (lipemic index), and genes directly related to ASP production, storage lipid metabolism and inflammation.

However, there are several limitations to this study. The samples were derived from morbidly obese subjects, limiting interpretations to that population. Further, although there were often large relative differences between the two groups (LAT vs HAT), the limited number of samples restricted the statistical power. There was also no functional analysis of the adipose tissue. On the other hand, the strengths of the study are that no samples were pooled and microarray analysis was conducted individually on all 21 samples. It should be noted that intact adipose tissue was specifically chosen for the analysis. This tissue represents a pool of adipocytes as well as preadipocytes, macrophages, and endothelial cells. Direct analysis of the tissue prevented the introduction of artefactual changes resulting from processing and separating the various cell populations. Hence, this can be viewed as both a strength and a weakness.

Interestingly, while the design of the study allowed for a comprehensive comparison of gene expression between SC and OM adipose tissue, we discovered that the significant differences were found primarily in the SC tissue. SAM analysis determined that OM tissue did not have significant differences between the LAT and HAT groups. Targeted analysis of specific biological pathways allowed us to compare genes from many different gene families simultaneously. Differential results between LAT and HAT in the SC tissue included genes involved in the alternative complement system, lipid synthesis, lipolysis, oxidation, fatty acid binding proteins, markers of differentiation, pro-inflammatory, anti-inflammatory, macrophage recruitment, and matrix remodelling genes. This contrasts markedly with a previous study comparing insulin resistant obese with insulin sensitive obese where differences were primarily noted in OM tissue, with no differences in SC tissue [12]. This is a particularly interesting point. While differences in omental adipose tissue in relationship to insulin resistance have been noted (in microarray studies and others), our present study specifically identified differences in subcutaneous adipose tissue with respect to ASP related metabolism. While differences in subcutaneous adipose tissue have been identified using other biochemical parameters (such as differences in triglyceride synthesis in subcutaneous adipose tissue in non-obese vs obese subjects) this is the first to identify this based on ASP metabolism. We speculate (as a hypothesis to be tested) that this may indicate a compensatory mechanism, whereby genes related to ASP are concomitantly increased with genes related to lipid storage, however this remains to be tested in future studies.

In contrast to the demonstrated changes in the gene families described above, there were no significant differences in housekeeping genes, insulin signalling genes and non-related complement genes between these two groups (LAT vs HAT). We demonstrated that four genes commonly reported as housekeeping genes [30], B2M, GUSB, PPIA, and TFRC, were not significantly different between the LAT and HAT groups nor did they correlate with age or BMI. Further, not only were plasma glucose and insulin comparable between the 2 groups, the expression of genes related to insulin resistance and insulin signalling such as insulin receptor InsR, IRS-1, IRS-4, IGF1, and IGF2 were comparably expressed between LAT and HAT groups, and did not correlate with age or BMI. Further, the expression of other complement genes (C5) and receptors (C3aR, C5aR) that are not involved in ASP production or signalling were not different between groups (LAT and HAT). This suggests that there is selective regulation of metabolic genes directly related to the role of ASP, independent of insulin or other immune related genes.

On the other hand, there was a strong association between increased plasma ASP and TG (lipemic index) and expression of the genes related directly to ASP production and function. Thus complement C3, factor B and adipsin were generally increased in the microarray in HAT vs LAT groups. Further, C5L2, the receptor for ASP, was assessed by real-time PCR, and expression was also increased in HAT vs LAT. C5L2, a G protein coupled receptor, has been shown to be present on human adipocytes and preadipocytes [17], but this is the first quantitative evaluation of C5L2 reported in human adipose tissue. While C5L2 is increased in the HAT group, the expression of C5L2 in the LAT group is very similar to the non-obese control group in both SC and OM tissue. Further, in the present study there was a significant correlation between C5L2 expression in SC and OM adipose tissue. These results are consistent with increased binding of ASP to adipose plasma membranes in obese subjects vs non-obese subjects, although the C5L2 receptor was as yet unrecognized at that time, and no plasma data was available [47]. We have also previously demonstrated an association between SC and OM adipose tissue for radiolabelled ASP binding to plasma membranes, across a range of BMI from obese and non-obese subjects. On the other hand, in our previous study, ASP binding and affinity was greater in SC vs OM adipose tissue, while in the present study, C5L2 mRNA expression was not different in SC vs OM, which could reflect a further level of regulation at the plasma membrane.

This is the first study evaluating expression of ASP related genes, including the receptor C5L2, in the context of plasma ASP. The few studies that have examined complement C3 gene expression in adipose tissue have demonstrated associations with BMI, adipose tissue site, insulin sensitivity, age and postprandial lipemia [48-53]. Only one study examined the levels of plasma ASP in relationship to adipose tissue gene expression and demonstrated significant associations between C3 mRNA, plasma ASP and both insulin sensitivity and postprandial TG [51].

This raises the question whether chronically increased plasma ASP represents an ASP resistant state (analogous to increased plasma insulin reflecting insulin resistance), or whether the increased ASP is compensatory. Hypothetically, the presence of increased plasma ASP, increased expression of C3, factor B and adipsin in the microarray and increased C5L2 based on real-time PCR would suggest a compensatory mechanism, indicative of a more effective response, and interpretation of the results are evaluated in that context below.

There was a striking correlation between the three genes most relevant to ASP production: C3, Factor B, and adipsin (aka Factor D), constituting an ASP triad. Other complement genes (C3aR and C5aR) that are neither related to ASP production nor signalling do not correlate with the "ASP triad." This selectivity within the alternative complement pathway and the correlation with many metabolic genes points to the physiological relevance. A general trend observed was that the HAT group has up-regulated genes for lipid TG storage. Along with the high plasma ASP and high C5L2 receptor, there are multiple genes including intracellular lipid storage, lipoprotein lipase, and fatty acid trafficking genes that are increased in the HAT group. The simultaneous increase in both lipid storage and lipolytic genes suggests an overall increase in substrate cycling and fatty acid flux [2,54]. Concurrently, de novo TG synthesis and oxidative genes were down-regulated. Taken together, the cells appear to be responding to an increased plasma TG level in the HAT group and are increasing their uptake and storage of TG. Many of the increased storage genes correlated with the "ASP triad" of genes including DB1 and LPL. Among the oxidative genes, ACC correlates negatively with the ASP triad.

Changes in adipose tissue function implicate alterations in differentiation genes, remodelling genes, and inflammatory complications [40,41,55,56]. The HAT group is characterized by increased in differentiation factors CEBPα, β, and CEBPδ as well as PPAR γ, consistent with the increased TG storage genes. Interestingly, we have previously demonstrated that ASP stimulates adipogenesis in cultured 3T3-L1 and 3T3-F442A cells, reflected by early increases in CEBP, followed later by increases in PPARγ, DGAT-1, adipsin and TG accumulation [57].

The HAT group is also characterized by increased anti-inflammatory gene TGFβ1 and inhibitors of metalloproteinases TIMP1, TIMP3 and TIMP4 and decreased metalloproteinase MMP20 and MMP10. On the other hand, there were few increases in pro-inflammatory genes, although pro-inflammatory and cell remodelling factors such as PIG7, CCR2 and MMP2 were increased. An interesting link between the role of the alternative complement pathway factors ASP and C5L2 and immune and adipose have been supported by active demonstration of adipose tissue macrophage infiltration, apoptosis and tissue remodelling in obesity, and the differences demonstrated between HAT and LAT.

## Conclusion

Overall, the profile of C5L2 receptor, ASP gene expression and metabolic factors in adipose tissue from the HAT group of morbidly obese subjects suggests a compensatory response associated with the increased plasma ASP and TG. A shift in the various pro-inflammatory, anti-inflammatory, macrophage recruitment and matrix remodelling proteins demonstrates a differential expression in the HAT group, although the specific benefits or deleterious effects of this regulation remain to be elucidated.

## Methods

### Subjects

Men and women living in Montreal, Canada were recruited for this study. Altogether, for the microarray there were two men and nine women, all morbidly obese (BMI > 40 kg/m^2^). Chi^2 ^analysis indicated no significant difference in gender distribution. A sample of subcutaneous and omental adipose tissue along with a fasting blood sample were collected during gastric bypass surgery (n = 11). Ethics approval for this project was obtained from the McGill University Health Centre (Royal Victoria Hospital) ethics review committee (Montreal, QC) and subjects signed an informed consent form prior to participation. Of the 11 subjects, none had previously been identified as having diabetes or any other known disease (other than the presence of morbid obesity). All women were pre-menopausal. None were taking hypoglycaemic or lipid lowering medication. Body weight was stable at the time of study.

For the real time RT-PCR analysis, a lean control group was included to compare C5L2 gene expression (a gene not on the microarray) to the morbidly obese subjects. This group consisted of four non-obese subjects, two women and two men. Adipose tissue samples were collected during elective hysterectomy surgery (n = 2), valve replacement (n = 1), and hernia (n = 1) surgeries. All other inclusion/exclusion parameters were the same as for the morbidly obese group. Although we cannot rule out the presence of an inflammatory component in these subjects, all were elective and not emergency surgeries.

### Blood samples

Venous blood samples were collected in the fasting state into non-heparinized EDTA tubes. Samples were collected at 7:00 am, on the ward following a 12 hour fast, prior to transfer of patients to surgery. Blood samples were centrifuged at 2000 rpm at 4°C for 10 minutes and plasma was stored at -80°C for later analysis. Insulin levels were measured by RIA (Medicorp, Montreal, Canada). Glucose was measured by colorimetric enzyme assay (GOD-PAP, Roche Diagnostics. Plasma triglycerides were measured by GPO-PAP (glycerol phosphate oxidase coupled to phenol and 4-aminophenazone) method. ASP levels were measured by colorimetric enzymatic ELISA [44].

### Calculation of Indices

A lipemic index utilizing the fasting values of ASP and TG was calculated. ASP strongly correlates with postprandial TG [22], therefore a lipemic index of [ASP × TG] may be indicative of ASP effects on clearance of postprandial TG.

### Adipose tissue microarray

Adipose tissue was collected at the beginning of the operation (within 30 minutes) into sterile 50 mL conical tubes and immediately flash- frozen in liquid nitrogen. Subcutaneous adipose tissue was collected from the subcutaneous abdominal wall and omental adipose tissue was collected from the greater omentum which is representative of intra-abdominal adipose depots drained by the portal vein. RNA was extracted from tissues using Qiagen spin columns (Qiagen, Mississauga, Canada) following manufacturer's protocol for fatty tissue with the addition of two RNase free DNase treatments (Qiagen) between RW1 washes. RNA quality was assessed by spectrometry (260/280 ratio) with ratios of 1.9 - 2.0. As well, RNA quality was assessed by chromatography. Synthesis of cRNA by in vitro transcription and hybridisation was performed as previously described [12,58]. Briefly, 10 ug of high quality total RNA was converted to cDNA and then to biotin-labelled cRNA (10 mM Biotin-11-UTP, Perkin-Elmer) by linear amplification (iExpress Assay reagent kit, GE Healthcare Bio-Sciences, Montreal, Quebec). 10 ug of labelled cRNA was hybridised to CodeLink UniSet Human 20 K I (GE Healthcare Bio-Sciences). The microarray slides were processed according to manufacturer's instructions and analysed using Codelink System Software (GE Healthcare Bio-Sciences). The spot intensities were median normalized (signal intensity of probe/median intensity of all discovery probes). The results have been deposited to GEO database under accession number GSE 15524.

### Real Time RT-PCR

Real time RT-PCR was used to evaluate the expression pattern of C5L2, the ASP receptor, because no probes for C5L2 were present on the microarray. The specific primer/probe sets used were human C5L2 (Hs_00218495). Confirmatory RT-PCR was also analyzed for 7 additional genes (complement C3 (C3), C5a receptor (C5aR), CD36, diacylglycerol acyltransferase 2 (DGAT2), fatty acid binding protein 4 (FABP4), CEBPγ and insulin receptor (INSR), as well as a housekeeping gene beta-2-microglobulin (B2M, 4326319E). All primeréprobe sets were obtained from Applied Biosystems (Toronto, Canada). RNA was collected as described above for the real time RT-PCR and run separately from the microarray. RNA (2 ug) was reverse transcribed to cDNA using the High Capacity cDNA RT kit (Applied Biosystems) according to manufacturer's protocols. All primer/probe sets were validated over an exponential range of 1:2 to 1:7500 dilutions of cDNA concentrations. A positive control consisting of pooled adipose tissue RNA isolated from human adipose tissue was used to establish the dynamic range of the assay for each primer/probe specifically in adipose tissue. Detection of the fluorescent signal was quantified by Rotor-Gene 3000 (Corbett Research). The control sample was assayed in each real-time PCR run to ensure consistency. For all samples, a dilution of 1:50 of the cDNA was used, which was linear within the tested exponential range, and all samples (n = 15) were analysed simultaneously, in duplicate or triplicate (no samples were pooled). Expression patterns for all genes, including C5L2 were analysed by real-time PCR using delta delta C_T _method expressed relative to the housekeeping gene B2M, according to manufacturer's protocol. Further, B2M was shown to be consistent across all samples (no significant changes) in both the microarray analysis (Additional File 2, Table S2) and in real time RT-PCR.

### Analysis and Statistics

Plasma data is presented as means ± SEM, and the two groups (HAT vs LAT) were compared by 2-sided t-test. The results were considered significant with p-values ≤ 0.05. For real-time PCR, data is presented as means ± SEM data. For C5L2 data, the groups were compared by two-way ANOVA for tissue (SC and OM) and groups (HAT, LAT, and NO) with all-pairwise post-hoc test used to compare significant differences between groups. For all other genes analyzed by real-time PCR, the two groups (HAT vs LAT) were compared by 2-sided t-test. The results were considered significant with p-values ≤ 0.05.

Two types of analysis were conducted for the microarray analysis: (i) SAM analysis and (ii) targeted analysis of biologic pathways involved in lipid storage and inflammation. For the former, analysis was conducted using SAM (Significant Analysis of Microarrays) program [29] version 3.09 and available at http://www-stat.stanford.edu/~tibs/SAM using suggested guidelines (see on-site manual). This program is a validated statistical technique for identifying differentially expressed genes across high density microarrays. This analysis technique provides a list of significant differentially expressed genes and their q values, and an estimate of the false discovery rate (FDR) which represents the percentage of genes that could be identified by chance [29]. Following SAM analysis of subcutaneous and omental tissues, we further analyzed the data targeting specific metabolic biologic pathways (according to the a priori hypothesis).

For the targeted microarray analysis, the following procedure was used: (i) using known biochemical pathways, a list of proteins/genes involved in ASP generation, complement related factors, lipid storage metabolism including TG synthesis, lipolysis and oxidation, adipocyte differentiation, and inflammatory genes were collated; (ii) accession numbers were identified using Entrez Gene http://www.ncbi.nlm.nih.gov/; (iii) the Codelink gene list was screened to tag all of the accession numbers present in the array; (iv) all genes identified with the associated SC and OM datasets were then pooled and (v) the data was then statistically analyzed for HAT vs LAT. All data was assembled prior to initiating analysis on HAT vs LAT. All genes identified in these pathways are presented for SC and OM tissues (Additional File 1, Table S1), whether significantly different or not (according to a 2-sided t-test).

Microarray data were expressed using mean ± SEM. The cross-nested design was to analyze two experimental factors: (i) the comparison between two groups (LAT versus HAT), and (ii) between the different tissues (SC vs OM). A mixed model analysis was performed with interaction between the fixed factors. To proceed with the analysis, we used a model with a compound symmetry structure. For most variables, values were log transformed to stabilize variances. Reported p values are based on these transformations. Relationships between variables were expressed using Spearman correlation coefficients. For all microarray data, the results were considered significant with p-values ≤ 0.025, where ns indicates not significant. Graphical and statistical analyses were conducted using GraphPad Prism (San Diego, CA), SAS, version 9.1.3 (SAS Institute Inc, Cary, NC) and SigmaStat (Jandel Scientific, CA).

## Competing interests

The authors declare that they have no competing interests.

## Authors' contributions

RM designed the overall project, recruited samples, carried out the microarray analysis and real-time PCR analysis as well as analyzed the data and drafted the manuscript. WC and HLL were responsible for microarray analysis and real-time PCR (with RM), as well as contributing to data analysis and writing of the manuscript. SS, a biostatistician, was responsible for statistical analysis, interpretation and contribution to writing the manuscript. KC designed and oversaw the research as principal investigator and contributed to writing the manuscript. All authors read and approved the final manuscript.

## Pre-publication history

The pre-publication history for this paper can be accessed here:

http://www.biomedcentral.com/1755-8794/3/3/prepub

## Supplementary Material

Additional file 1 **Table 1: Microarray expression in subcutaneous and omental adipose tissue of genes involved in adipose metabolism**. Table of results of subcutaneous and omental adipose tissue in LAT and HAT, and correlation values with selected parameters Click here for file

Additional file 2 **Table 2: Housekeeping genes and genes related to insulin resistance**. Table of results of housekeeping genes in LAT and HAT Click here for file

Additional file 3 **Figure 1: Expression of oxidation related genes in SC adipose tissue in LAT and HAT**. Graphics figure of correlations between oxidation related genes Click here for file

Additional file 4 **Figure 2: Expression of fatty acid binding protein genes in SC adipose tissue in LAT and HAT**. Graphics figure of correlations between fatty acid binding protein genes Click here for file

Additional file 5 **Figure 3: Inflammatory profile of pro- and anti-inflammatory genes in SC adipose tissue in LAT and HAT**. Graphics figure of correlations between pro- and anti-inflammatory genes Click here for file

Additional file 6 **Figure 4: Macrophage recruitment and matrix metalloproteinase genes in SC tissue in HAT and LAT**. Graphics figure of correlations between macrophage recruitment and matrix metalloproteinase genes Click here for file
