# Supplementary figures and images for: Association of adipocyte genes with ASP expression: a microarray analysis of subcutaneous and omental adipose tissue in morbidly obese subjects
Source: BMC Med Genomics. 2010 Jan 27;3:3. doi: 10.1186/1755-8794-3-3 (PMC2843642; doi:10.1186/1755-8794-3-3)

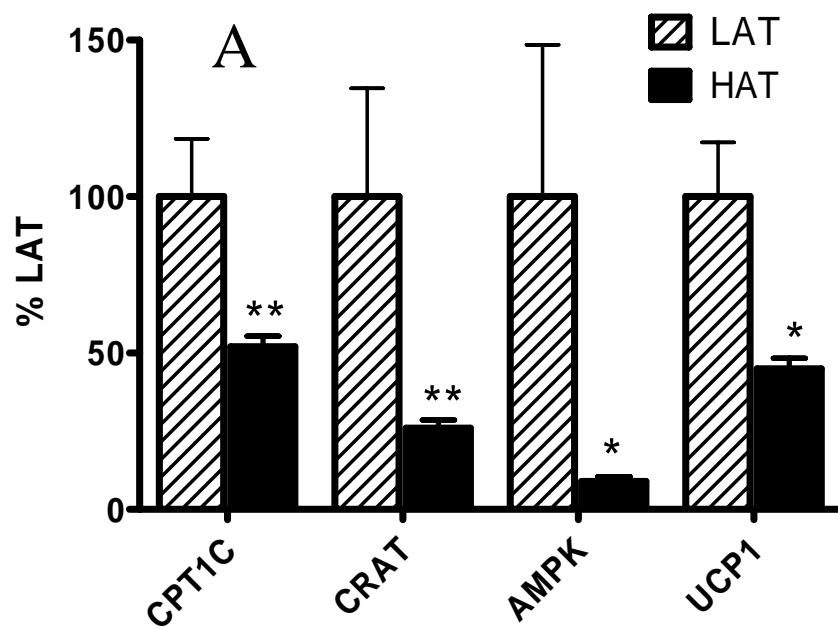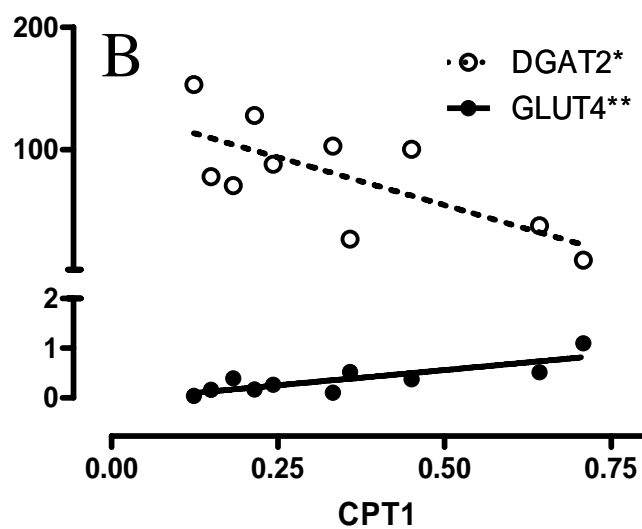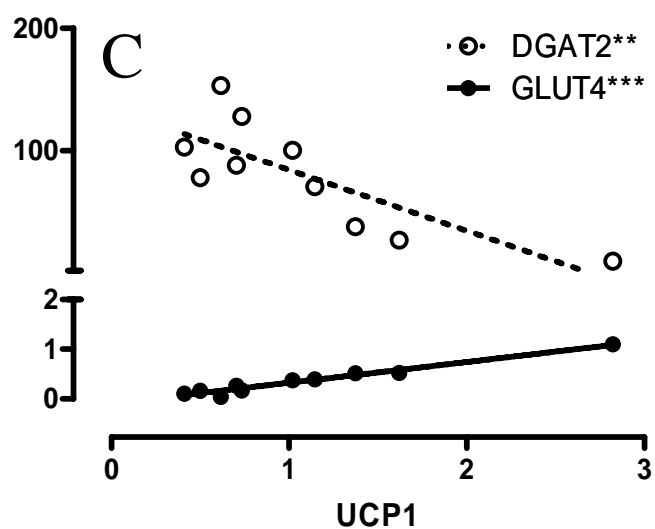

Supplementary Figure 1

Supplement: Additional file 1 — Table 1: Microarray expression in subcutaneous and omental adipose tissue of genes involved in adipose metabolism. Table of results of subcutaneous and omental adipose tissue in LAT and HAT, and correlation values with selected parameters [file 1755-8794-3-3-S1.PDF]

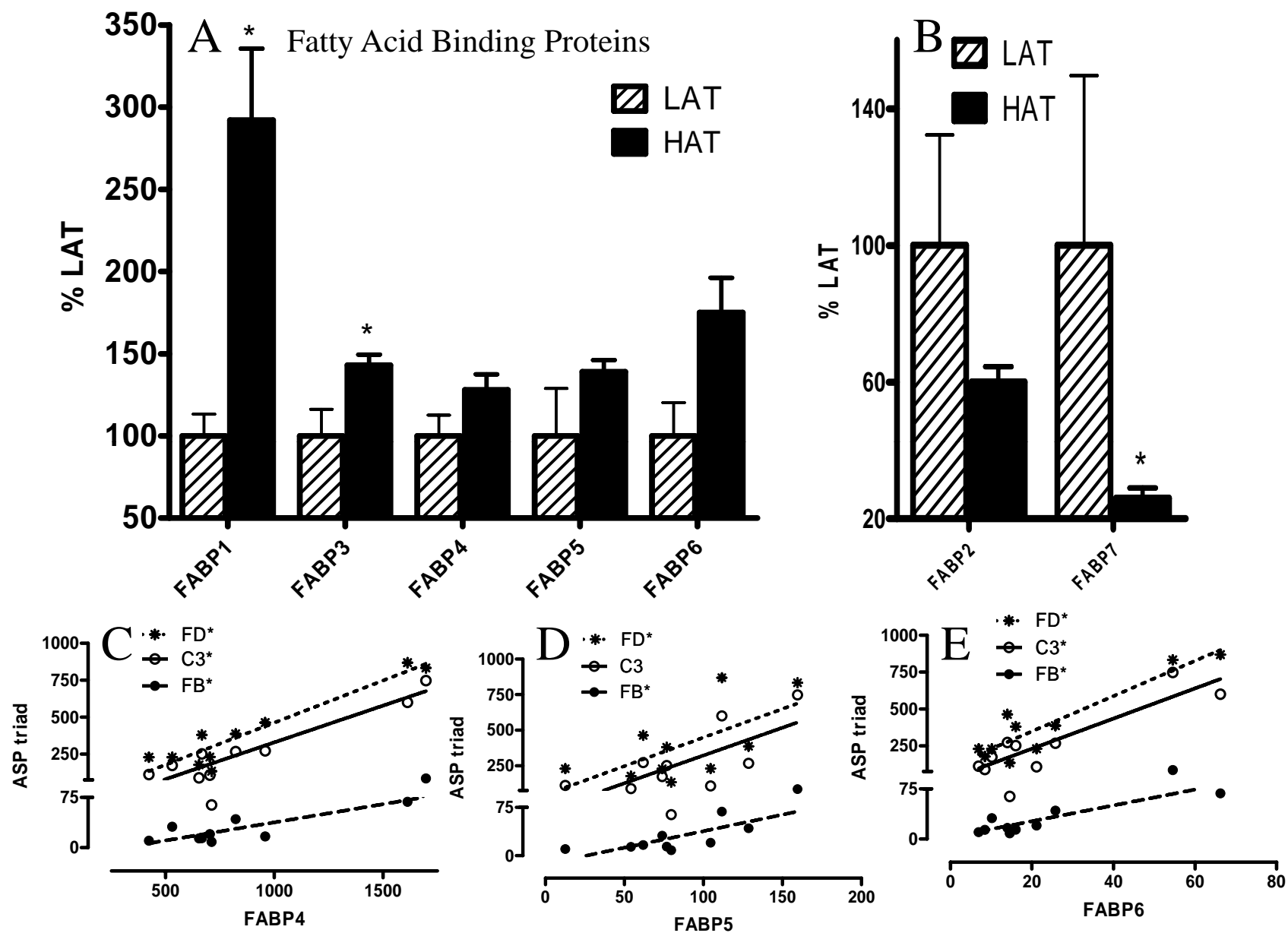

Supplementary Figure 2

Supplement: Additional file 2 — Table 2: Housekeeping genes and genes related to insulin resistance. Table of results of housekeeping genes in LAT and HAT [file 1755-8794-3-3-S2.PDF]

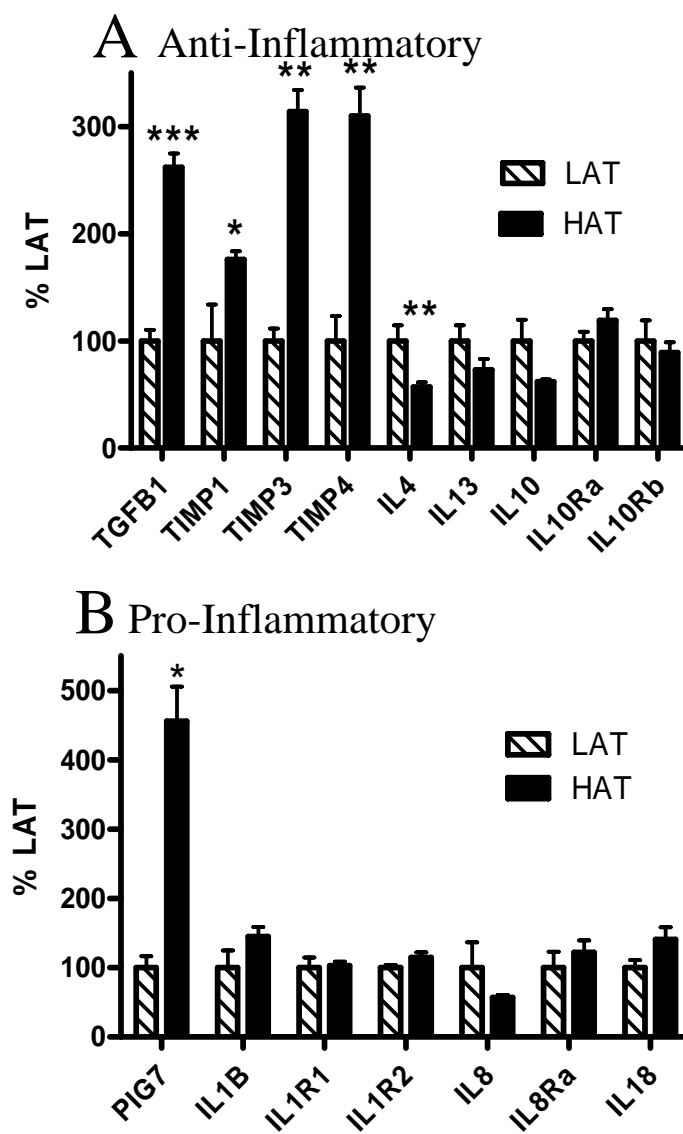

Supplementary Figure 3

Supplement: Additional file 3 — Figure 1: Expression of oxidation related genes in SC adipose tissue in LAT and HAT. Graphics figure of correlations between oxidation related genes [file 1755-8794-3-3-S3.PDF]

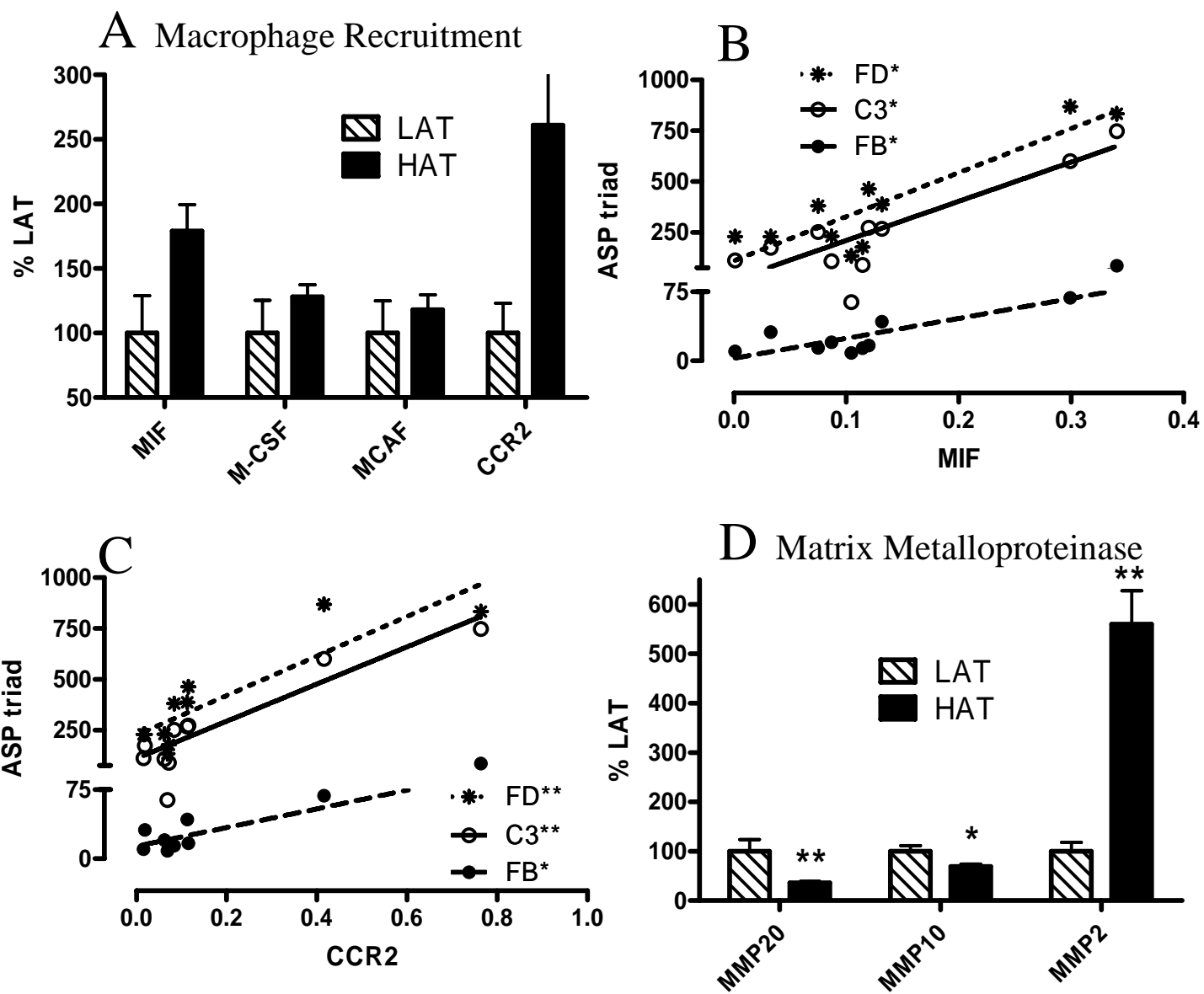

Supplementary Figure 4

Supplement: Additional file 4 — Figure 2: Expression of fatty acid binding protein genes in SC adipose tissue in LAT and HAT. Graphics figure of correlations between fatty acid binding protein genes [file 1755-8794-3-3-S4.PDF]
