# Supplementary material for: Association of adipocyte genes with ASP expression: a microarray analysis of subcutaneous and omental adipose tissue in morbidly obese subjects
Source: BMC Med Genomics. 2010 Jan 27;3:3. doi: 10.1186/1755-8794-3-3 (PMC2843642; doi:10.1186/1755-8794-3-3)
Supplement: Additional file 5 — Figure 3: Inflammatory profile of pro- and anti-inflammatory genes in SC adipose tissue in LAT and HAT. Graphics figure of correlations between pro- and anti-inflammatory genes [file 1755-8794-3-3-S5.PDF]

Supplementary Table 1: Microarray Expression in Subcutaneous and Omental Adipose Tissue and Spearman Correlations of Selected Genes in Subcutaneous Adipose Tissue

**Gene**	**ACCN**	**SC-LAT**	**SC-HAT**	**OM-LAT**	**OM-HAT**	**BMI**	**ASP**	**FB**	**C3**	**Adip**	**CD36**	**Cebpγ**	**HSL**	**Glut4**	**Dgat2**	**UCP1**	**Pparδ**	**Mmp20**
**ASP production related genes**																		
C3	NM_000064	217± 52	290± 103	1060± 292	481± 163			0.806 **		0.939 ***			-0.697 *	0.648 *				
Factor B	NM_001710	23.2± 9.8	34.5± 11.4	238± 66	109± 47*	0.685 *			0.806 **	0.770 **		0.709 **						
Adipsin (FD)	NM_001928	359± 69	407± 118	384± 49	288± 59			0.770 **	0.939 ***				-0.612 *	0.661 *		0.612 *		
Factor H	NM_000186	11.8± 5.9	19.2± 4.3*	7.91± 2.88	7.56± 2.0											-0.709 **	-0.636 *	
Factor I	NM_000204	0.584± 0.239	0.673± 0.086	0.69± 0.13	1.23± 0.34					-0.600 *								
Properdin	NM_002621	5.5± 1.0	10.1± 3.4	14.0± 5.5	9.2± 1.6	0.636 *		0.952 ***	0.794 **	0.709 *		0.721 **						
CPN1	NM_001308	0.171± 0.046	0.153± 0.040	0.27± 0.13	0.22± 0.07			0.479	0.612 *	0.661 *		0.697 *						
CPD	NM_001304	0.714± 0.046	0.492± 0.038**	0.89± 0.24	0.59± 0.11						-0.770 **	-0.636 *						
CR1	NM_000651	1.71± 0.80	0.277± 0.074***	0.47± 0.33	0.25± 0.09		-0.600 *							0.612 *		0.697 *	0.709 *	0.745 *
**Complement Related Factors**						BMI	ASP	FB	C3	Adip	CD36	Cebpγ	HSL	Glut4	Dgat2	UCP1	Pparδ	Mmp20
C3aR	NM_004054	0.899± 0.230	1.35± 0.38	0.47± 0.21	0.80± 0.21													
C5	NM_001735	0.210± 0.115	0.325± 0.080	0.38± 0.17	0.33± 0.02			-0.818 ***	-0.915 ***	-0.903 ***				-0.624 *				
C5aR	NM_001736	0.140± 0.024	0.109± 0.044	0.22± 0.12	0.27± 0.09													
**Lipid Synthetic Genes**						BMI	ASP	FB	C3	Adip	CD36	Cebpγ	HSL	Glut4	Dgat2	UCP1	Pparδ	Mmp20
CD36	NM_000072	137± 35	367± 83**	219± 59	211± 42		0.636 *					0.624 *						

DB1	NM_020548	163± 55	263± 35*	238± 16	202± 41	0.661 *		0.639 *	0.548	0.509	0.770 **	0.782 **						
DGAT1	NM_012079	2.18± 0.73	5.37± 0.93***	2.25± 0.62	2.55± 0.35				-0.661 *	-0.600 *			0.988 ***	-0.964 ***	0.952 ***	-0.867 ***	-0.770 **	-0.891 ***
DGAT2	NM_032564	39.6± 17.5	96.8± 15.0***	24.3± 9.9	34.9± 5.4								0.915 ***	-0.891 ***		-0.770 **	-0.673 *	-0.794 **
PAP2A2	AF014403	45.7± 15.4	116± 22**	74.7± 14.5	94.7± 22.8	0.624 *					0.939 ***	0.661 *						
SCD1	AB032261	1.61± 0.64	16.9± 3.9***	5.9± 2.8	12.8± 3.6								0.927 ***	-0.952 ***	0.903 ***	-0.879 ***	-0.830 ***	-0.915 ***
ME1	NM_002395	0.550± 0.194	0.868± 0.167*	0.30± 0.04	0.59± 0.08*				-0.721 *	-0.867 ***								
FASN	NM_004104	0.460± 0.306	1.99± 0.89*	0.51± 0.06	0.98± 0.30								0.830 ***	-0.782 **	0.830 ***	-0.673 *	-0.673 *	-0.709 *
GLUT4	NM_001042	0.668± 0.218	0.235± 0.063***	0.31± 0.14	0.35± 0.11				0.648 *	0.661 *			-0.952 ***		-0.891 ***	0.952 ***	0.758 **	0.879 ***
ELOVL2	NM_017770	45.1± 8.5	21.8± 1.7***	38.1± 11.9	41.9± 10.6											0.758 **	0.782 **	0.624 *
ELOVL4	NM_022726	1.45± 0.63	266± 0.039***	0.51± 0.33	0.38± 0.03						-0.721 *	-0.636 *						
**Lipolysis Related Genes Extracellular**						BMI	ASP	FB	C3	Adip	CD36	Cebpγ	HSL	Glut4	Dgat2	UCP1	Pparδ	Mmp20
LPL	NM_000237	235± 80	436± 102*	302± 40	294± 56+			0.600 *	0.430	0.564 *		0.782 **						
PNLIP	NM_000936	1.32± 0.68	0.198± 0.046**	0.98± 0.85	0.27± 0.08							-0.650 *						
LIPC	NM_000236	1.42± 0.51	2.08± 0.67	1.70± 0.64	0.80± 0.20*		0.648 *	0.842 ***	0.673 *	0.709 *		0.879 ***						
EL	NM_006033	0.235± 0.088	0.117± 0.048*	0.06± 0.03	0.07± 0.030.												0.842 ***	0.782 **
**Lipolysis Related Genes Intracellular**						BMI	ASP	FB	C3	Adip	CD36	Cebpγ	HSL	Glut4	Dgat2	UCP1	Pparδ	Mmp20
MGLL	NM_007283	39.6± 20.1	71.2± 12.1*	30.3± 9.4	34.5± 7.1								0.758 **	-0.733 **		-0.794 **	-0.770 **	-0.806 **
CES1	NM_001266	1.91± 0.16	7.01± 1.66**	2.16± 0.78	3.36± 0.72					-0.648 *			0.709 *	-0.782 **		-0.794 **	-0.806 **	-0.830 **
Adiponutrin	NM_025225	4.06±	2.53±	3.19±	2.88±				0.661	0.697			-0.867	0.915	-0.685	0.939	0.782	0.867

		0.90	0.60*	0.54	0.49				*	*			***	***	*	***	**	***
HSL	NM_005357	7.99± 2.00	25.3± 5.7***	14.3± 4.2	13.9± 2.0				-0.697 *	-0.612 *				-0.952 ***	0.915 ***	-0.855 ***	-0.733 *	-0.855 ***
Perilipin	NM_002666	283± 146	501± 54**	262± 44	322± 62								0.636 *	-0.564	0.515			
**Oxidation Related Genes**						BMI	ASP	FB	C3	Adip	CD36	Cebpγ	HSL	Glut4	Dgat2	UCP1	Pparδ	Mmp20
ACC	X68968	1.25± 0.27	2.85± 0.63*	2.74± 1.14	3.39± 1.10			-0.591	-0.782 **	-0.782 **			0.891 ***	-0.915 ***	0.697 *	-0.903 ***	-0.685 *	-0.806 **
CPT1C	NM_152359	0.511± 0.165	0.267± 0.045**	0.54± 0.15	0.48± 0.10				0.600 *				-0.794 **	0.745 **	-0.600 *	0.661 *		0.721 *
CPT2	NM_000098	0.572± 0.266	0.866± 0.102*	0.48± 0.11	0.55± 0.10													
CRAT	NM_004003	0.335± 0.200	0.087± 0.024**	0.17± 0.06	0.32± 0.13								-0.709 *	0.806 **		0.891 ***	0.867 ***	0.842 ***
AMPK	NM_006252	2.49± 2.10	0.213± 0.092*	0.34± 0.13	0.23± 0.06				0.636 *	0.636 *			-0.733 *	0.758 **		0.806 **	0.733 **	0.818 **
PRKAA1	NM_006251	0.349± 0.045	0.636± 0.102*	0.39± 0.14	0.42± 0.10													
PRKAB2	NM_005399	1.61± 0.46	0.810± 0.152*	0.94± 0.21	0.76± 0.13								-0.782 **	0.855 ***	-0.673 *	0.952 ***	0.879 ***	0.867 ***
PRKAG1	NM_002733	0.271± 0.051	1.23± 0.27**	0.41± 0.15	0.78± 0.15*					-0.648 *	0.612 *		0.697 *	-0.745 **		-0.697 *	-0.661 *	-0.721 *
PRKAG2	AF087875	0.203± 0.152	0.488± 0.054*	0.35± 0.05	0.46± 0.05													
PRKAG3	NM_017431	1.06± 0.55	0.371± 0.072*	0.50± 0.09	0.56± 0.16		-0.661 *							0.685 *		0.794 **		
UCP1	U28480	1.78± 0.53	0.800± 0.155*	0.96± 0.29	0.75± 0.07					0.612 *			-0.855 ***	0.952 ***	-0.770 **		0.806 **	0.855 ***
UCP2	NM_003355	9.77± 1.32	11.67± 1.52	12.3± 2.8	11.2± 1.3				0.612 *									
UCP3	NM_022803	0.986± 0.241	0.882± 0.147	1.01± 0.29	0.95± 0.15				0.600 *	0.673 *				0.685 *		0.721 *		0.721 *
UCP4	NM_004277	0.254± 0.137	0.204± 0.053	0.26± 0.09	0.22± 0.03													
**Fatty Acid Binding Proteins**						BMI	ASP	FB	C3	Adip	CD36	Cebpγ	HSL	Glut4	Dgat2	UCP1	Pparδ	Mmp20

FABP1	NM_001443	0.137± 0.032	0.401± 0.158*	0.11± 0.04	0.18± 0.07							0.806 **						
FABP2	NM_000134	0.322± 0.180	0.194± 0.039	0.42± 0.27	0.24± 0.07													
FABP3	NM_004102	0.834± 0.231	1.19± 0.14*	0.82± 0.05	0.78± 0.09				-0.600 *				0.818 **	-0.794 **	0.648 *	-0.733 *		-0.685 *
FABP4	NM_001442	734± 160	939± 186	935± 99	864± 160			0.624 *	0.685 *	0.745 *		0.661 *						
FABP5	NM_001444	67.7± 33.6	94.3± 13.1	76.4± 16.8	68.5± 10.8			0.733 *	0.503	0.552 *		0.867 ***						
FABP6	NM_001445	15.6± 5.5	27.3± 8.8	23.3± 3.6	23.8± 4.9			0.745 *	0.612 *	0.721 *		0.891 ***						
FABP7	NM_001446	0.228± 0.196	0.059± 0.017*	0.25± 0.11	0.23± 0.07													
**Adipose Tissue Differentiation**						BMI	ASP	FB	C3	Adip	CD36	Cebpγ	HSL	Glut4	Dgat2	UCP1	Pparδ	Mmp20
CEBPα	NM_004364	8.26± 3.87	28.6± 5.6**	11.3± 4.8	14.7± 3.1				-0.685 *	-0.600 *			0.964 ***	-0.915 ***	0.830 ***	-0.842 ***	-0.661 *	-0.745 *
CEBPβ	NM_005194	0.406± 0.207	2.34± 0.89**	0.81± 0.27	1.60± 0.45				-0.830 ***	-0.830 ***			0.830 ***	-0.855 ***	0.782 **	-0.782 **	-0.697 *	-0.806 **
CEBPδ	NM_005195	0.353± 0.096	0.564± 0.217	0.44± 0.21	0.58± 0.13													
CEBPε	NM_001805	0.319± 0.107	0.180± 0.038*	0.28± 0.15	0.22± 0.10								-0.745 *	0.673 *	-0.685 *			0.685 *
CEBPγ	NM_001806	10.83± 2.75	15.52± 3.87	16.8± 1.94	12.6± 2.52		0.612 *	0.709 *	0.527	0.698 *	0.624 *							
PPARα	NM_--5036	0.673± 0.164	0.671± 0.050	0.45± 0.12	0.50± 0.09													
PPARδ	NM_006238	1.21± 0.37	0.509± 0.057**	0.75± 0.27	0.55± 0.09								-0.733 **	0.758 **	-0.673 *	0.806 **	0.939 ***	-0.636 *
PPARγ	NM_--5037	39.2± 23.4	77.1± 12.5**	44.2± 10.8	48.3± 9.3			-0.636 *	-0.915 ***	-0.903 ***			0.830 ***	-0.806 **	0.612 *	-0.733 *	-0.648 *	-0.782 **
PPARγCoA	NM_013261	0.604± 0.434	0.173± 0.031*	0.31± 0.15	0.22± 0.07													
**Anti-Inflammatory Genes**						BMI	ASP	FB	C3	Adipn	CD36	Cebpγ	HSL	Glut4	Dgat2	UCP1	Pparδ	Mmp20
TGFB1	NM_000660	0.935±	2.45±	1.78±	1.61±								0.661	-0.685	0.721	-0.770	-0.830	-0.721

		0.172	0.31***	0.69	0.19								*	*	*	**	***	**
TIMP1	NM_003254	26.1± 15.4	45.8± 5.2*	72.1± 12.0	79.7± 14.5	0.758 **	0.855 ***									-0.648 *		
TIMP3	NM_000362	1.43± 0.29	4.51± 0.75**	1.97± 0.52	2.86± 0.62						0.624 *		0.867 ***	-0.915 ***	0.770 **	-0.903 ***	-0.636 *	-0.745 **
TIMP4	NM_003256	6.47± 2.56	20.1± 4.5**	11.9± 5.1	14.6± 4.1				-0.758 **	-0.733 **			0.952 ***	-0.976 ***	0.842 ***	-0.927 ***	-0.673 *	-0.806 **
IL4	NM_000589	6.01± 1.49	3.42± 0.72**	4.21± 0.83	3.79± 0.79				0.612 *	0.661 *			-0.855 ***	0.927 ***	-0.697 *	0.976 ***	0.782 **	0.842 ***
IL13	NM_002188	0.272± 0.067	0.200± 0.074	0.25± 0.04	0.22± 0.15			0.685 *	0.927 ***	0.964 ***			-0.733 **	0.782 **	-0.600 *	0.721 *		0.709 *
IL10	NM_000572	0.933± 1.10	0.578± 0.051	0.55± 0.17	0.56± 0.07													
IL10Ra	NM_001558	1.42± 0.21	1.686± 0.401	1.06± 0.26	1.31± 0.20		0.661 *											
IL10Rb	NM_000628	12.3± 4.1	10.9± 3.2	25.3± 5.5	15.0± 3.8			0.879 ***	0.964 ***	0.952 ***		0.648 *						
**Pro-Inflammatory Genes**						BMI	ASP	FB	C3	Adip	CD36	Cebpγ	HSL	Glut4	Dgat2	UCP1	Pparδ	Mmp20
PIG7	NM_004862	3.23± 0.92	14.7± 4.2**	8.74± 3.65	10.81± 3.06				-0.745 **	-0.758 **			0.927 ***	-0.976 ***	0.842 ***	-0.927 ***	-0.721 **	-0.867 ***
IL1B	NM_000576	2.58± 1.10	3.74± 0.96	1.35± 0.25	3.43± 1.01													
IL1R1	NM_000877	2.20± 0.57	2.25± 0.34	4.94± 1.51	3.44± 0.46													
IL1R2	NM_004633	2.14± 0.14	2.46± 0.41	2.70± 0.27	2.54± 0.30			0.673 *	0.915 ***	0.806 **								
IL8	AF385628	2.15± 1.37	1.24± 0.21	0.78± 0.12	0.89± 0.14	0.697 *		0.915 ***	0.685 *	0.673 *		0.685 *						
IL8Ra	NM_000634	1.50± 0.60	1.83± 0.70	1.05± 0.25	2.45± 1.03			0.685 *	0.661 *	0.624 *								
IL18	NM_001562	0.506± 0.098	0.712± 0.231	0.65± 0.28	1.49± 0.34													
**Macrophage recruitment**						BMI	ASP	FB	C3	Adip	CD36	Cebpγ	HSL	Glut4	Dgat2	UCP1	Pparδ	Mmp20
MIF	L19686	0.084± 0.042	0.150± 0.045	0.13± 0.03	0.13± 0.04			0.636 *	0.624 *	0.661 *		0.709 *						

M-CSF	NM_000757	0.185± 0.081	0.237± 0.046	0.19± 0.02	0.19± 0.11										0.612 *			
MCAF	M24545	9.52± 4.08	11.2± 3.0	13.6± 4.36	8.18± 2.06													
CCR2	NM_000647	0.081± 0.033	0.212± 0.105	0.13± 0.05	0.29± 0.13			0.624 *	0.782 **	0.794 **		0.709 *						
**Matrix Metalloproteinase Genes**						BMI	ASP	FB	C3	Adipn	CD36	Cebpγ	HSL	Glut4	Dgat2	UCP1	Pparδ	Mmp20
MMP20	NM_004771	1.22 ± 0.50	0.445 ± .083**	0.46± 0.09	0.71± 0.11								-0.855 ***	0.879 ***	-0.794 **	0.855 ***	0.939 ***	
MMP10	NM_002425	19.2 ± 3.8	13.3 ± 2.4*	14.8± 2.8	13.8± 2.1					0.673 *			-0.818 **	0.915 ***	-0.685 *	0.964 ***	0.806 **	0.864 ***
MMP2	NM_004530	0.461 ± 0.144	2.58 ± 0.82**	0.61± 0.12	1.44± 0.47				-0.673 *	-0.758 **			0.612 *	-0.685 *		-0.782 **	-0.818 **	-0.782 **

Data presented are gene expression analysis of subcutaneous and omental adipose tissue from low ASP and TG obese subjects (LAT, n=4) and high ASP and TG obese subjects (HAT, n=7 analysed by microarray. The associated accession number (ACCN) is reported. Mean and standard deviation are presented for each group (all units are arbitrary), compared by t test with significant p values indicated as * $p < 0.05$; ** $p < 0.01$; *** $p < 0.001$. Spearman correlations (r value) for subcutaneous adipose tissue only between each gene and selected genes of interest are provided where r value is given for all correlations with $p<0.1$, and significance is indicated as * $p < 0.05$; ** $p < 0.01$; *** $p < 0.001$.
