# Supplementary material for: Association of adipocyte genes with ASP expression: a microarray analysis of subcutaneous and omental adipose tissue in morbidly obese subjects
Source: BMC Med Genomics. 2010 Jan 27;3:3. doi: 10.1186/1755-8794-3-3 (PMC2843642; doi:10.1186/1755-8794-3-3)
Supplement: Additional file 6 — Figure 4: Macrophage recruitment and matrix metalloproteinase genes in SC tissue in HAT and LAT. Graphics figure of correlations between macrophage recruitment and matrix metalloproteinase genes [file 1755-8794-3-3-S6.PDF]

Supplementary Table 2: Microarray Internal Controls: Housekeeping Genes and Genes Related to Insulin Resistance

**Gene**	**ACCN**	**SC**			**OM**		
		**LAT**	**HAT**	**t-test**	**LAT**	**HAT**	**t-test**
**Housekeeping Genes**							
B2M	NM_004048	89.2 ± 49.6	113.4 ± 12.6	ns	99.1 ± 19.4	99.5 ± 17.5	ns
GUSB	NM_000181	0.498 ± 0.166	0.665 ± 0.268	ns	0.436 ± 0.095	0.651 ± 0.159	ns
PPIA	NM_021130	18.2 ± 8.7	23.4 ± 3.2	ns	23.7 ± 7.1	19.1 ± 3.5	ns
TFRC	NM_003234	0.227 ± 0.078	0.211 ± 0.035	ns	0.339 ± 0.163	0.298 ± 0.044	ns
**Insulin Resistance Related Genes**							
InsR	X02160	7.66 ± 1.03	8.59 ± 3.28	ns	16.38 ± 4.62	10.17 ± 2.88	ns
IRS1	NM_005544	4.67 ± 1.29	5.67 ± 1.54	ns	7.33 ± 1.56	5.56 ± 1.17	ns
IRS4	NM_003604	4.84 ± 0.72	3.06 ± 0.91	ns	3.17 ± 0.47	3.57 ± 0.85	ns
IGF1	NM_000618	8.02 ± 3.46	11.74 ± 2.33	ns	5.67 ± 1.64	7.30 ± 1.10	ns
IGF2	NM_000612	0.845 ± 0.147	1.194 ± 0.305	ns	1.373 ± 0.816	1.076 ± 0.232	ns

Data are means ± standard deviation, where B2M beta-2-microglobulin, GUSB beta glucuronidase, InsR insulin receptor, IRS insulin receptor substrate, PPIA peptidyl prolyl isomerise A, and TFRC transferrin receptor. Data were analyzed by t test where p ns indicate not significant.

Supplementary Figure Legends.

Supplementary Figure 1: Expression of Oxidation related genes in LAT and HAT. Expression of oxidation genes (A) CPT1C, CRAT, AMPK, and UCP1 in SC adipose tissue from LAT (hatched bars) and HAT (solid bars) as assessed by microarray where LAT expression is set at 100%. Correlation between metabolic genes GLUT4 and DGAT2 with (B) CPT1 and (D) UCP1 shown as GLUT4 (solid circles) and DGAT2 (stars). R values (Spearman coefficient) for GLUT4 and DGAT2, respectively are (B) 0.745, -0.600 and (C) 0.952, -0.770. Lines are based on linear regression. Data is expressed as means ± SEM where * $p<0.025$, ** $p<0.01$ and *** $p<0.001$.

Supplementary Figure 2: Expression of Fatty Acid Binding Protein genes in LAT and HAT. Gene expression of fatty acid binding protein genes (A) FABP1, 3, 4, 5 and 6 and (B) FABP2 and 7 in SC adipose tissue from LAT (hatched bars) and HAT (solid bars) as assessed by microarray where LAT expression is set at 100%. Correlation between the ASP triad of genes and (C) FABP4, (D) FABP5, and (E) FABP6 are shown for C3 (open circles), FB (solid circles), and adipsin (stars). R values (Spearman coefficient) for C3, FB, and adipsin respectively, are (C) 0.684, 0.624, 0.745, (D) 0.503, 0.733, 0.552 and (E) 0.612, 0.745, 0.721. Lines are based on linear regression. Data is expressed as means ± SEM where * $p<0.025$, ** $p<0.01$ and *** $p<0.001$.

Supplementary Figure 3: Inflammatory profile of pro and anti-inflammatory genes in HAT vs LAT group. Gene expression of anti (A) and pro (B) inflammatory genes in SC adipose tissue from LAT (hatched bars) and HAT (solid bars) as assessed by microarray where LAT expression is set at 100%. Data is expressed as means ± SEM where * $p<0.025$, ** $p<0.01$ and *** $p<0.001$.

Supplementary Figure 4: Changes in Macrophage recruitment and Matrix Metalloproteinase genes in HAT vs LAT groups; correlations with ASP triad. Gene expression of macrophage recruitment genes (A) and matrix metalloproteinase genes (B) in SC adipose tissue from LAT (hatched bars) and HAT (solid bars) as assessed by microarray where LAT expression is set at 100%. Correlation between the ASP triad of genes and (B) MIF and (C) CCR2 are shown as C3 (open circles), FB (solid circles), and adipsin (stars). R values (Spearman coefficient) for C3, FB, and adipsin respectively, are (B) 0.624, 0.636, 0.661 and (C) 0.782, 0.624, 0.794. Lines are based on linear regression. Data is expressed as means ± SEM where * $p<0.025$, ** $p<0.01$ and *** $p<0.001$.
